# The Interfunctional Relationship Between Theory of Mind and Private Speech

**DOI:** 10.11621/pir.2024.0101

**Published:** 2024-03-01

**Authors:** Leonardo Daniel Rivera Valdez, Vicente Arturo López Cortés

**Affiliations:** a *Monterrey Institute of Technology and Higher Education, Mexico City, Mexico*; 2 Meritorious Autonomous University of Puebla, Mexico

**Keywords:** theory of mind (ToM), private speech (PS), social cognition, internalization, cultural-historical, executive function, speech types (ST)

## Abstract

**Background:**

Theory of mind is the capacity to explain and predict the behavior of others. Charles Fernyhough’s dialogical model of psychological functions offers a vision of theory of mind that considers the social dimension and the importance of language, especially inner and private speech, for a person’s ability to represent and manipulate multiple perspectives, and its connection to executive function.

**Objective:**

There is little direct research on Fernyhough’s model. The present study addressed that gap by studying the relationship between theory of mind, private speech, and executive function (planning) in the preschool years.

**Design:**

Data were collected from a total of 86 preschool children from the Mexican state of Tlaxcala; 24 were from the first grade of preschool, 30 from the second, and 32 from the third. Their degree of internalization and their speech types (*i.e*., social and private) were obtained by coding their utterances during free play and during performance of a Tower of London task. Lastly, their theory of mind was assessed with a change of location and an unexpected contents task.

**Results:**

No correlation was found between children’s theory of mind and their degree of internalization. However, inaudible private speech was correlated (negatively) to theory of mind performance in the third grade of preschool. Furthermore, their scores on the Tower of London task were negatively correlated with social speech and positively correlated with inaudible private speech, while the reverse was true for time of execution.

**Conclusion:**

The results suggested that the degree of internalization is a measure of the regulative function of language, not the ability to manipulate perspectives, and that it is inadequate for capturing subtle differences between performance and speech types. Role-playing conditions were recommended as better tasks for exploring the capacities for manipulating and understanding different perspectives during the preschool years.

## Introduction

Theory of mind (ToM) is the capacity to explain or predict the behavior of others from inferring their mental states (Premack & Woodruff, 1978). Typically, children tend to pass tests associated with such a construct at well-established ages: different desires at two years old; different beliefs at three years old; knowledge access at four years old; false beliefs (FB) at five years old; real-apparent emotion at six years old; and second order FB tasks at seven (Perner & Wimmer, 1985; Wellman & Liu, 2004; but see Wellman et al., 2006 for different results).

Of relevance in the literature is the discussion of how to explain the fact that 4-5-year-old children are able to perform the FB tasks. These are a family of tasks where kids need to give an answer that is coherent with another person’s beliefs, even though the state of affairs of the world is different (Doherty, 2008; Perner et al., 1987; Wimmer & Perner, 1983). An example is the classic change of location task, where two characters are playing with an object, but one of the characters needs to leave the scene and leaves the object in box A. While this character is gone, the other character moves the object to box B. When the first character comes back to the scene, the experimenter asks some control questions to the participant (*e.g*., memory: where did the character leave the object?; reality: “where is the object right now?), and the key question “where will he search for the object?” (Wimmer & Perner, 1983). In order to answer correctly, the child needs to answer that he will search for the object in box A, since he left the object there, and had no way of knowing about the change of location (Dennett, 1978).

## Theories of Theory of Mind

These results are very well established, but the explanations for them are very controversial. The various theories that try to explain these results could be categorized as follows: 1) modular-nativist theories; 2) representational theories; 3) linguistic theories; 4) executive function theories; and 5) cultural-historical theories.

Modular-nativist models are theories that subscribe to modules (*i.e*., they are domain specific, encapsulated, mandatory, have a specific neural architectonic, and their development is innately specified; Fodor, 1983), and they have an evolutionary reason for their emergence (Baron-Cohen, 2005). For example, the model of Leslie (1987) explains that there is a module for representation that underlies the capacity for pretend play and the capacities for representing FB. In fact, the model proposes that the only reason why children still fail the FB task at four is because of its computational charge or, which is the same thing, because the task is too difficult in other not-yet-developed domains (*e.g*., executive functions; Fodor, 1992). Another model that follows the same train of thought is the implicit ToM model of Onishi & Baillargeon (2005), which explains the sensitivity for ToM as expressed by the looking-times in 15-month-old children. Finally, Baron-Cohen’s TESS (The Empathizing System) model (2005) incorporates, within a modular framework, the broad phenomena of social cognition, such as dyadic and triadic relations, emotion recognition, empathy, etc.

The representational models tend to posit that in order to perform the FB task, children need to grasp the concept of representation. This would enable them to comprehend that the representations that someone has from reality may differ from the actual state of the world (Perner, 1991). Leslie’s previously mentioned model (1987) is one of them, but the most relevant one is Perner’s model of meta-representation (1991). In this model, Perner proposes that secondary representations (*i.e*., a model of the world that refers to states of affairs that are not present) are enough to explain the data of pretend play, while Leslie (1987) postulates the need for meta-representations. Contrary to Leslie, Perner believes that there is no need to postulate such knowledge at that age, but it is necessary to postulate it when they pass the FB test. Such a conception of representation would enable children to notice that a representation is intentional to some states of affairs of the world, but that the truth value of such representation differs from the one describing the current state of the world (Perner, 1991).

On the other hand, linguistic theories refer to those theories that try to explain ToM development as a function of one dimension of language, or by emphasizing the properties of one dimension (*e.g*., syntax, semantics, pragmatics, etc.). These theories may differ in how strongly they conceptualize that dependence. Take, for example, the de Villers (2007) and de Villers & de Villers (2014) syntax determinism model. This model proposes that the development of the structure of syntax complementation enables children to produce and comprehend mental propositional structures such as “Juan believes that the object is in box A” which are key in the FB tasks. Having these structures would enable them to understand that the truth value of the whole sentence differs from the truth value of its complement: that the object is in box B while the subject believes, falsely, that it is in box A. Because of that, the authors suggest, children are able to pass the FB tests.

Then, we have the theories that try to explain the capacity to perform the FB task due to the development of executive functions such as inhibition. Executive function, as a general construct, is understood as the capacity to plan and control one’s current activity in accordance with a goal (Zelazo et al., 1997). This construct is understood to be composed of a subset of interrelated subfunctions (*i.e*., working memory, inhibition, and cognitive flexibility) that work together for the above-mentioned purpose (Zelazo, 2015). These family of theories say that in order to pass the FB tests, a child needs to inhibit the preponderant response (*i.e*., that the object is in box B), to give the answer according to the beliefs of others (i.e., that the object is in box A; Carlson & Moses, 2001; Carlson et al., 1998, 2002, 2004; Doherty, 2008; Perner & Lang, 1999). Because of that, the emphasis is placed not on ToM itself, but like Fodor and Leslie, on the associated computational capacities which do not allow its adequate expression.

Finally, and relevant to the purposes of this work, we have the cultural-historical theories. Such theories tend to subscribe to at least some basic postulates: 1) a genetic view of development; 2) that the origin of psychological functions is social; and 3) that inter-psychological and intra-psychological functions are mediated by signs and symbols (Fernyhough, 1996; Vygotsky, 2012d; Wertsch, 1993). Some authors may also subscribe to a functional systems approach to psychological functions (Fernyhough, 2010; Luria, 1974, 2002; Luriya & Artem’eva, 1970; Vygotsky, 2012a, 2012b).

A theory that invokes all these aspects is Fernyhough’s (1996, 2008, 2009) dialogic theory of psychological functions, which centers specifically on speech (*i.e.,* social, private, and inner speech). Fernyhough (1996, 2009) starts from Vygotsky’s (2012d) notion of the genetic law of development that establishes that psychological functions appear two times in development. First, they appear as inter-psychological functions (on the social plane), and later as intra-psychological functions (on an individual plane).

However, as Fernyhough (2009) points out, Vygotsky never developed the dialogical consequences of this particular aspect of his theory. The fact that the intra-psychological plane is derived ontogenetically from the inter-psychological plane seems to imply that multiple perspectives are to be internalized from a context where dialogue is the norm. Moreover, that it would have dialogical properties, *i.e*., turn-taking structure (especially at the beginning of the internalization process) and/or multiple perspectives represented in it. These dialogical properties would enable a person to represent multiple perspectives (*i.e.,* a position-bound orientation towards a state of affairs, perceptual or not), at the same time.

For example, in a common sentence like “I love tacos, but my mother says they are not that good,” two different perspectives are represented (*i.e.,* my perspective and my mother’s perspective about tacos); this would have an origin in the conversation with my mom. At a later stage we might develop a more abbreviated form of the previously mentioned sentence like “Love tacos…not that good” where both subjects are evident for the subject (therefore are omitted), syntax is reduced, and meaning is condensed in this simpler, but not well-formed, sentence (Fernyhough, 2008; Wertsch, 1993). This is possible, in Vygotsky’s view (2012c), because in the process of internalization, structural and functional changes occur in the internalization of language. If it all starts in dialogue, private speech (PS; *i.e.,* audible speech referring to oneself) plays a linking role between dialogue and inner speech (*i.e.,* silent speech to oneself). For Vygotsky (2012c), PS is not merely an epiphenomenon of the child’s activity; on the contrary, for him, it plays a regulative and planning role in activity (*i.e*., what we now call executive function). Moreover, it undergoes several processes of syntactic abbreviation (*i.e.,* predicative form) and semantic agglutination (*i.e*., an influx of sense where meanings are fused). The above-mentioned characteristics enable us to represent multiple perspectives at the same time, while the cognitive charge of the process is simplified, especially when it changes from a complete dialogue to synthesized dialogue, where the perspectives become agglutinated (Fernyhough, 2008, 2009).

This capacity to manipulate multiple perspectives is for Fernyhough (2008) what enables children to perform the FB task. This is because to perform such tasks, you need to derive conclusions while you maintain both perspectives in mind. Therefore, the author believes that the general ability of being able to manipulate multiple perspectives at the same time, is needed to pass the FB test. In fact, in a previous study, it was found that PS had a quadratic relationship to ToM (*i.e*., to have first a positive relationship to ToM, then no relationship, and finally a negative relationship). It found a positive correlation between ToM and PS in children between 3 and 4 years old; no correlation between ToM and PS at 4–5 years old; and a negative correlation at 5–6 years old (Fernyhough & Meins, 2009). But there is not a ton of direct research in this topic, since the study was the only one that was carried out with a typically developed population and was limited to children of the English Midlands.

Finally, as Alderson-Day and Fernyhough (2015) have noted more recently, and would be evident from the above discussion, another important aspect of their theory is that PS has some properties that seem not to be dialogic. As Luria and Vygotsky mentioned, it also has regulative functions (Luria, 1961; 2000; Vygotsky, 2012c). These properties have also been mentioned by authors such as Zelazo and Baddeley for whom PS plays a role in maintaining information in working memory which is crucial for maintaining goals and rules for coordinating action (Baddeley, 1992; 2010; Zelazo, 2004; 2015; Zelazo et al. 1998). In fact, Alderson-Day and Fernyhough (2015) believe that such PS and inner speech are not dialogical but monological. This means that this type of speech lacks the properties of dialogue (*e.g*., turn taking, or having multiple perspectives) but is directed to oneself and does not need to consider others. Because of these considerations, we should also expect to find a relationship between PS and executive function. In fact, that is what the literature has reported in studies of planning, cognitive flexibility, and working memory (Alarcón-Rubio et al., 2014; Benigno et al., 2011; Fatzer & Robers 2012; Fernyhough & Fradley, 2005).

## The Present Study

Given the lack of direct research in this line of work, we tried to replicate Fernyhough & Meins’ (2009) study, but we expanded it by adding the analyses of executive functioning (*i.e*., planning; see below), since many authors have pointed out that there is an intimate relationship between these processes (Baddeley, 1992; Alderson-Day & Fernyhough, 2015; Zelazo, 2004; 2015; Zelazo et al., 1998).

First, we hypothesized that ToM would be related to PS scores differently according to age (*i.e*., positive in younger children and negative in older children). This is because if PS is to be understood as a general capacity for the manipulation of multiple perspectives, then the internalization process would follow the quadratic relationship (see above) which was suggested by Fernyhough and found in the above-mentioned study (Fernyhough & Meins, 2009).

Second, we hypothesized that ToM and the internalization process (*i.e*., the emergence of more mature forms of speech regulation; see below) would be different across the groups (*i.e.,* preschool grades), with the scores being higher as children got older.

Finally, we expected that different phases of the internalization process would relate differently to executive function: specifically, that more mature forms of speech regulation (*i.e*., social<PS<inner) would be associated with better performance in planning (*i.e*., time of execution and scores in a planning task; see below).

## Methods

### Participants

Participants in the study were recruited from the preschools “Jardín de Niños General Lázaro Cárdenas del Río” and “Jardín de Niños Salvador Díaz Mirón” in the state of Tlaxcala, Mexico. They were included if they did not have previous signs of a neurological condition, or a learning problem reported by their teachers or parents. Consent was obtained from their parents, and the children and their parents were free to withdraw from the study at any time. Of 91 children, four left the study, and one was discontinued because his teacher said that he was receiving language therapy. The total sample thus consisted of 86 participants: 24 of them were from the first grade of preschool (boys = 9 and girls = 15; mean age = 4 years, SD = .257; range: 3.50–4.33 years); 30 from the second grade of preschool (boys = 13 and girls = 17; mean age = 5.02 years, SD = .311; range: 4.58–5.41 years); and 32 from the third grade of preschool (boys = 14 and girls = 18; mean age = 5.98 years, SD = .279; range: 5.58–6.75 years).

### Materials

#### Private Speech Coding

The coding scheme was adopted from Fernyhough & Meins (2009), where videotaped sessions were divided into utterances according to temporal and semantic criteria: temporally by units of 2 seconds of difference, and semantically by changes in the theme of the utterance. Then, the children’s utterances were divided according to whether they were social or private. An utterance was considered social if: 1) there was visual contact between the participant and another person for at least 2 seconds while the utterance was produced; 2) if contact (*e.g*., physical) occurred between the participant and another person for at least 2 seconds while the utterance was produced; 3) the content of the utterance mentioned explicitly the previous utterance of another person; or 4) temporarily the utterance followed with a time-lapse of less than 2 seconds the previous utterance of another person. All the utterances that did not fulfill these conditions were considered private speech (PS) (Winsler, 2009; Winsler et al., 2005).

PS utterances were coded following Berk’s classification (1986). Level 1 was when the PS (PS1) was irrelevant to the task; it was word play or repetition; expressed emotions irrelevant to the task; or was directed to imaginary characters or non-human characters. Level 2 was when the PS (PS2) was relevant to task; described child’s own activity or were self-guiding commentaries; were self-answered questions; or expressed emotions relevant to the task. Level 3 (PS3) was when it was externalized inner speech relevant to the task (*e.g*., verbal murmurs, whispers, and lip or tongue movements). Lastly, a degree of internalization measure, as in the original study, was computed by summing the total amount of PS2 and PS3, and dividing it by the amount of time in minutes $\frac{TotalPS2+TotalPS3}{Time(\min)}$ Fernyhough & Meins, 2009), taken to complete the task.

Then, for the analyses of other types of speech (*i.e.,* social and subtypes of PS), since the times of codification were different for every child, we divided the frequencies of speech by the amount of time, *i.e*., $\frac{TotalSpeechType}{Time(\min)}$ (Winsler, 2009). Finally, for finer analyses, following Winsler (2009), we classified the dominant speech type (ST) as a function of the dominant social or PS type: 1) If social speech was dominant, then social ST was coded; 2) if PS1 was dominant, then non-relevant audible ST was coded; 3) if PS2 was dominant, then relevant audible ST was coded; and 4) if PS3 was the dominant type, then inaudible ST was coded. We also introduced an internal ST category to deal with the cases where no speech was produced. Whenever a discrepancy occurred, such that two ST were dominant, then the least developed one was coded to avoid inflating the participant’s capacity, since the previous form of regulation was manifested to the same degree. For example, if relevant audible and inaudible ST were dominant, then the former was used.

The transcription and coding of the videotapes were made by L.D. Rivera Valdez. Recordings were visualized in an MSI computer with the relevant software and listened to with headphones for more clarity. To evaluate the inter-rater reliability, L trained and paid an individual who was unaware of the hypotheses. This person coded 25% of the transcripts (21 children). The measured degree of agreement for the social/private speech was almost perfect with a 99% of agreement (Kappa = .971, p<.001), while the classification for the different types of private speech also had a near perfect agreement of 96% (Kappa = .913, p<.001).

#### Free Play

As in Fernyhough & Meins’s study (2009), since the group of first graders was very young, we followed them by recording the free play sessions in groups of four kids for about 16 minutes maximum. Two cameras were positioned in a silent room to capture different angles of the room. Their speech was coded following the above-mentioned coding schemas.

#### Tower of London

As Fernyhough and Fradeley (2005) and Fernyhough and Meins (2009) did, we gave the Tower of London (ToL) test to the second and third graders. The ToL task consists of three pegs and three rings of different colors (*e.g*., blue, red, and green), one copy for the participant, and the others for the researcher to create a model as the target of the trial. The experimenter told the participant that “you need to make sure that your toy looks equal to this one (the model),” and presented them with four different levels (*i.e*., 2, 3, 4 and 5 moves) of the task. Then, the participants were told that: 1) they should use one hand only; 2) that they could not move more than one ring at a time; 3) that they could not leave the ring on the table and then move another ring; and 5) they should always put the ring on a peg first and then move another one. Lastly, the children were told that “Some children like to talk out loud when they resolve this task; if you want, you can talk.” This was done to encourage the children to talk; otherwise, they might not talk even if it would be helpful for them. After all the instructions were mentioned, the researcher had a practice trial (of two moves) with the participant to be sure that the rules were clear, and any doubt was clarified. The session was recorded and coded as specified above.

In addition, the time of execution for every level and the total score were considered for analysis. A point was granted for every level they passed with the minimum number of moves (*e.g*., 2 for two moves, 3 for three moves, etc.), and the point was granted only if the orientation proportioned to the child was limited to the clarification of the rules of the task. A maximum of 5 points total was possible.

#### False Belief Tasks

Following Perner et al. (1987), we also administered a change of location test and a version of the false contents test. The change of location task (as described above) was presented in a format of scenes while the researcher was narrating and pointing to the relevant elements of the story. Three questions were asked: 1) a memory question to see if the children remembered where the character left the object; 2) a reality question to see if they knew the current location of the object; and 3) the FB question to see if they could respond that the character would check the box where she left the ball. Children were granted two points if they responded correctly to the FB question and the memory and reality questions; otherwise, no point was granted.

In the false contents task, a chocolate milk bottle with water inside was employed instead of Smarties or Lunetas (the Mexican version of the candy). Such an object was chosen by previously testing some objects with various kids and by asking the teachers about the most convenient one to use. The children were introduced to the chocolate milk bottle and were asked, “What is this? What does it contain?” After they gave the wrong answer (chocolate milk), the true content (water) was revealed. Then, three questions were asked: a reality question to see if they knew what truly was in the bottle after they had made the mistake (*i.e.,* said that there was water); a question about their own false beliefs to see if they knew what their previous belief was before the revelation (chocolate milk) of the real content (water); and a FB question to see if they could infer that a person who saw the chocolate milk bottle for the first time would have a FB as they did (*i.e*., they would say that it contains chocolate milk). A point was granted if children answered correctly to the reality question and their own belief question, and two additional points were granted if they correctly answered the reality question and the FB question.

The points scored on both tasks were summed, and a total ToM score was computed with a maximum score of 5 points if they answered correctly to the control questions, to their own belief question, and to both FB questions.

### Data Analysis

First, Shapiro-Wilk tests were run to check for normality for all variables. Since the data was not normally distributed, we employed non-parametric tests. Second, Kendall’s correlations were computed for ToM, degree of internalization, speech as a function of time, and ToL variables, for every preschool year. Lastly, robust ANOVAS with trimmed means were performed for the differences in ToM, degree of internalization, and ST across groups, and for times and scores of the ToL for every ST.

## Results

### Descriptive Statistics

*Table 1* shows the descriptive statistics of ToM and degree of internalization, as well as the types of private speech as a function of time in minutes for every preschool grade. *Table 2* shows the frequencies of the ST. In general, it can be shown that the first graders still had a predominantly social form of regulation. Meanwhile, the second graders still had a predominantly social type of regulation, but other forms like PS2 or PS3 were becoming more dominant. Lastly, the third graders changed drastically, with PS3 becoming the dominant form of regulation, PS2 going to second place, and the social form of regulation third. On the other hand, ToM and degree of internalization become more developed from first to third grade.

**Table 1:** Descriptive Statistics for ToM and Degree of Internalization

	Grade	Social (min)	PS1 (min)	PS2 (min)	PS3 (min)	Degree of Internalization	ToM
N	1	24	24	24	24	24	24
	2	30	30	30	30	30	30
	3	32	32	32	32	32	32
Mean	1	1.40	.0653	.255	.00667	.262	1.58
	2	1.72	.00	.739	1.45	2.19	2.27
	3	.664	.0313	1.49	1.60	3.09	3.00
Standard deviation	1	.949	.154	.202	.0327	.207	1.44
	2	1.69	0.00	1.39	1.86	2.07	1.28
	3	1.17	0.177	1.69	1.66	2.07	1.37

**Table 2:** Frequencies of ST

ST	Grade	Frequencies	% of Total	Cumulative %
Social Speech	Preschool 1	22	25.6 %	25.6 %
	Preschool 2	14	16.3 %	41.9 %
	Preschool 3	5	5.8 %	47.7 %
Relevant Audible Speech	Preschool 1	2	2.3 %	50.0 %
	Preschool 2	5	5.8 %	55.8 %
	Preschool 3	8	9.3 %	65.1 %
Inaudible Speech	Preschool 1	0	0.0 %	65.1 %
	Preschool 2	10	11.6 %	76.7 %
	Preschool 3	14	16.3 %	93.0 %
Mental	Preschool 1	0	0.0 %	93.0 %
	Preschool 2	1	1.2 %	94.2 %
	Preschool 3	5	5.8 %	100.0 %

*Note: non-relevant audible speech was omitted since only one participant had it as a dominant ST*

### The Relationship Between ToM and Degree of Internalization

It was found that the relationship between ToM and degree of internalization was significant when group distinctions were ignored (τ = .198, p = .018). Then, correlations for every group were computed for the degree of internalization and the frequencies of speech as a function of time (see *Tables 4–6*). For ToM and degree of internalization, the first graders had a positive correlation which was not significant (τ = .155, p = .345); the second graders had a small positive correlation which was also not significant (τ = .164, p = .263); and finally, the third graders had a negative correlation which was not significant (τ = –.224, p = .151). Therefore, the quadratic relationship was present, but the relationships were not significant.

**Table 4:** Correlation Matrix Preschool 1 ToM and Degree of Internalization

		ToM	Social (min)	PS1 (min)	PS2 (min)	PS3 (min)	DI
ToM	Kendall’s Tau B	–					
	p-value	–					
Social (min)	Kendall’s Tau B	–.105	–				
	p-value	.515	–				
PS1 (min)	Kendall’s Tau B	–.041	.000	–			
	p-value	.820	1.000	–			
PS2 (min)	Kendall’s Tau B	.103	.154	.206	–		
	p-value	.530	.305	.222	–		
PS3 (min)	Kendall’s Tau B	.302	–.240	–.124	.091	–	
	p-value	.113	.170	.529	.611	–	
Degree of Internalization	Kendall’s Tau B	.155	.117	.180	.958	.234	–
	p-value	.345	.438	.287	< .001	.191	–

**Table 5:** Correlation Matrix Preschool 2 ToM and Degree of Internalization

		ToM	Social (min)	PS2 (min)	PS3 (min)	Degree of Internalization
ToM	Kendall’s Tau B	–				
	p-value	–				
Social (min)	Kendall’s Tau B	–.129	–			
	p-value	.382	–			
PS2 (min)	Kendall’s Tau B	.023	.025	–		
	p-value	.884	.860	–		
PS3 (min)	Kendall’s Tau B	.149	–.178	–.146	–	
	p-value	.318	.192	.312	–	
Degree of Internalization	Kendall’s Tau B	.164	–.036	.264	.675	–
	p-value	.263	.786	.061	< .001	–

**Table 6:** Correlation Matrix Preschool 3 ToM and Degree of Internalization

		ToM	Social (min)	PS1 (min)	PS2 (min)	PS3 (min)	Degree of Internalization
ToM	Kendall’s Tau B	–					
	p-value	–					
Social (min)	Kendall’s Tau B	–.224	–				
	p-value	.151	–				
PS1 (min)	Kendall’s Tau B	.254	.138	–			
	p-value	.132	.406	–			
PS2 (min)	Kendall’s Tau B	.183	.348	.152	–		
	p-value	.218	.018	.336	–		
PS3 (min)	Kendall’s Tau B	–.295	–.003	–.060	–.150	–	
	p-value	.044	.984	.699	.274	–	
Degree of Internalization	Kendall’s Tau B	–.224	1.000	.138	.348	–.003	–
	p-value	.151	< .001	.406	.018	.984	–

When we considered the frequencies of speech type as a function of time (in min), the results changed (*see Tables 4–6*). For the group of first and second graders, there were no significant correlations between the speech types of regulation and their ToM (p>.05). On the contrary, for the third grade, as expected, there was a significant negative correlation with PS3 (τ = –.295, p = .044).

While the degree of internalization was not satisfactory, the speech as a function of time was partially satisfactory for capturing the different relationships between ToM and the process of internalization. This was probably an effect of the measurement itself (degree of internalization), since it grouped together PS2 and PS3, while the other considered them separately.

### The Relationship Between the Degree of Internalization and Executive Function

We explored the relation of ToL variables (time and score; see *Table 7*) and the variables of internalization (see *Table 8*). The relationship between the degree of internalization and time of execution in the ToL for the groups of preschool was significant for the second and third graders, showing that as the degree of internalization increased, the time of execution was shorter (τ = –.279, p = .002). Yet, the degree of internalization and ToL scores were not correlated (τ = .113, p = .249). These results were also true when we considered the amount of speech in relation to time (in min), but some new patterns were revealed. Concretely, the points scored on the ToL test were negatively correlated with social speech (τ = –.376, p<.01) and had a tendency for a positive correlation with PS3 (τ = .178, p = .07). With respect to time the reverse was true; we observed a positive correlation with social speech (τ = .469, p<.01) and a negative correlation with PS3 (τ = –.218, p = .017). The analyses showed that as the internalization process matured, the regulatory capacities got better, as reflected by a reduction in ToL times and an increase in ToL scores.

**Table 7:** ToL Points and ToL Times for Preschool 2 and 3

	Grade	ToL Points	ToL Times
Mean	2	2.43	3.03
	3	2.84	1.45
Standard Deviation	2	1.14	2.05
	3	0.920	0.929

**Table 8:** Correlation Matrix Preschool 2 and 3 ToL and Degree of Internalization

		Points ToL	Time ToL	Social (min)	PS1 (min)	PS2 (min)	PS3 (min)	Degree of Internalization
Points ToL	Kendall’s Tau B	–						
	p-value	–						
Time ToL	Kendall’s Tau B	–.406	–					
	p-value	< .001	–					
Social (min)	Kendall’s Tau B	–.376	.469	–				
	p-value	< .001	< .001	–				
PS1 (min)	Kendall’s Tau B	–.092	.042	.023	–			
	p-value	.432	.695	.836	–			
PS2 (min)	Kendall’s Tau B	–.040	–.054	.116	.138	–		
	p-value	.701	.565	.243	.224	–		
PS3 (min)	Kendall’s Tau B	.178	–.218	–.084	–.034	–.118	–	
	p-value	.076	.017	.383	.754	.225	–	
Degree of Internalization	Kendall’s Tau B	.113	–.279	.007	.087	.430	.542	–
	p-value	.249	.002	.944	.416	< .001	< .001	–

### Differences in Degree of Internalization Across Preschool Grades

An ANOVA analysis was carried out with trimmed means at 20% to augment the study’s statistical power because the data were not normally distributed (Mair & Wilcox, 2020; Wilcox, 2017). The ANOVA with trimmed means showed that there were significant differences between the preschool groups in their degree of internalization (F = 25.1, p< .001). Post-hoc analyses were then conducted. They showed that the first graders had a lower degree of internalization than the second graders (ψ̂ = –1.52, p = .002); that the first graders had a lower degree of internalization than the third graders (ψ̂ = –2.85, p<.001); and that the second graders had a lower degree of internalization than the third graders (ψ̂ = –1.32, p = .035). Therefore, the degree of internalization consistently increased across the preschool years.

When we considered the speech type as a function of preschool group, trimmed mean ANOVAs were not possible, so Kruskall-Wallis ANOVAs were performed. The tests showed that there were significant differences for social speech (χ^2^ = 15.1, df = 2, p = .001), for PS1 (χ^2^ = 15, df = 2, p<.001), and PS3 (χ^2^ = 24.9, df = 2, p< .001), but not for PS2 (χ^2^ = 3.00, df = 2, p = .223). Post-hoc analyses revealed that the amount of social speech of the first graders was greater than that of third graders (W = –5.28, p< .01), and the second graders presented more social speech than the third graders too (W = –4.33, p = .006). For PS1, the first graders produced more than the second (W = 4.43, p = .005) and third graders (W = –3.71, p = .024). Finally, for PS3, the first graders produced less PS3 than the second (W = 6.585, p<.001) and third groups (W = 6.551, p<.001). This showed that social speech and PS1 became less significant through the preschool years, and more mature forms of regulation took the lead as the children got older (*i.e.,* PS3).

### Differences in ToM across Preschool Groups

An analysis of ANOVA with trimmed means for ToM scores was performed following the same logic as before. Significant differences were found across the preschool groups (F = 5.70, p<.008). Post-hoc analyses revealed that the first graders did not differ from the second group in ToM (ψ̂ = –0.785, p = .113); and that the first group had lower scores of ToM than the third ψ̂ = –1.612 p = .006); finally, the second group did not differ from the third (ψ̂ = –0.828, p = .113). The above showed that in general, ToM progressed from first to third grade, but that such progress was moderate since there were no differences between the first and second, nor second and third groups.

### Differences in Time and ToL Scores as a function of Speech Type

Finally, two ANOVA analyses with trimmed means were performed for the scores and times of the ToL as a function of the ST. The analyses indicated that there was a significant difference for the scores of the ToL (F = 3.34, p = .053). Post-hoc analyses showed that children who produced predominantly social ST showed lower scores on the ToL test than those who produced inaudible ST (ψ̂ = –1.063, p = .050), but did not differ from those who produced relevant audible ST (ψ̂ = –.444, p = .653), or an internal ST (ψ̂ = –1.250, p = .112). Moreover, those who produced more relevant audible ST did not differ from those with an inaudible ST (ψ̂ = –.618, p = .653) or internal ST (ψ̂ = –.806, p = .653). Finally, the group that produced more inaudible ST did not differ significantly from those who produced more internal ST (ψ̂ = –.188, p = .653).

Results of an ANOVA analysis of time as a function of the ST were significant too (F = 7.82, p = .002). The post-hoc analyses showed that those who produced more social ST took more time to resolve the ToL than those who produced relevant external ST (ψ̂ = 2.107, p = .013), an inaudible ST (ψ̂ = 2.225, p = .010), or internal ST (ψ̂ = 2.723, p = .003). Moreover, those who produced more relevant external ST did not differ from those with an inaudible ST (ψ̂ = .118, p = .588), or internal ST (ψ̂ = 0.616, p = .068). Finally, those with inaudible ST did not differ from those with an internal ST (ψ̂ = 4.98, p = .083).

The above analyses imply that, in general, in the change from social regulation to self-regulation, no matter which type of self-regulation, greater improvements in precision and speed emerge during the change from other to self-regulation.

## Discussion

In general, we found that the scores of ToM and degree of internalization did not correlate significantly across the preschool groups. Although they presented the positive correlation that was expected for first and second grades of preschool, the third grade of preschool had the expected negative correlation.

Yet, that was not the case when we considered speech as a function of time. With those variables, the relationships were specific to the speech type and were not mixed as in the degree of internalization since this is a variable that assumed that PS2 and PS3 are homogenous. For the group of first and second graders, there were no significant correlations between any speech type and ToM. But, for the third grade of preschool, there was a significant negative correlation between PS3 and ToM. The last result, and the previous results from the degree of internalization, are coherent with Fernyhough’s (2008) model, since we found that as the process of internalization matures, the manipulation of multiple perspectives would be so automatized that a regression to less mature forms would be detrimental to performance in ToM. This is because it would be a disadvantage to represent multiple perspectives in a less condensed form of representation, whereas (*ex hypothesi*) it is more costly in resources, since the elements of the functional system are not abbreviated, or sedimented, making the process less fluid and automatized.

Moreover, even though the relationships between ToM and degree of internalization were not significant, the differences across groups in their ToM and degree of internalization were clear. Specifically, the ToM of the first grade did not differ from the second grade, nor the second grade from the third grade, but it differed from the first grade to the third grade. This showed that in general, older children performed better in ToM compared to their younger peers. Nevertheless, it is important to note that the expected ages for passing the FB tests in previous studies (*i.e.,* 4–5 years) were not replicated with this sample from Tlaxcala. This is evident if we consider that the mean was about 3 out of 5 points even for children in third grade, and that the age range in this group was from 5.58 to 6.75 years (Doherty, 2008; Nilsson & López, 2016; Perner, 1991). These differences in development should be of great importance for those who study or work with the population of Mexico, and especially of Tlaxcala, but also very significant for the specialist in ToM, since it shows that there exist critical variations in the development of ToM across cultures. This, of course, may have affected the relationships between ToM and speech regulation.

Regarding the development of the degree of internalization, there was a well-marked difference across the preschool grades. The first graders had a lower degree of internalization than the second, and the second graders had a lower degree of internalization than the third-grade group, replicating previous results in the literature of PS (Fernyhough & Meins, 2009; Winsler, 2009). This was also true when their speech was measured as a function of time and ST. The children in first grade had more social speech and less mature forms of regulation, while the second and third graders had more mature forms of regulation (*i.e*., PS2 and PS3). With respect to the ST, this was reflected in the fact that in the first grade, social speech dominated. For the second grade, there was an important change; although social speech still dominated, other forms of regulation appeared, such as relevant audible speech and inaudible speech. Lastly, in the third grade group, social speech went to the background, and inaudible speech dominated. Moreover, internal speech appeared as a significant form of regulation.

As for executive functioning (*i.e*., planning), with respect to times, a negative correlation was found between the degree of internalization and the times of ToL, but not for the scores on the ToL. This was especially interesting relative to the ST used by the children. In specific, those who used social ST were slower in resolving the ToL test. This result conformed to Galperin’s (2021) conclusion that one of the most important changes in the development of psychological functions is the formation of the ideal or mental orientation (*i.e.,* when the orientation comes from within the child) of the action, as well as a process of automatization as internalization occurs. This was even clearer when we considered the frequency of speech type as a function of time (in min). It was clear that those who regulated themselves with social speech had poorer scores and took more time, while those who regulated themselves with PS3 acted faster and performed better. This is consistent with the literature, which has shown that PS is related to executive function performance in tasks of planning (Benigno et al., 2011; Fernyhough & Fradley, 2005), cognitive flexibility (Alarcón-Rubio et al., 2014), and working memory (Fatzer et al., 2012). This is also what we would expect within the Alderson-Day and Fernyhough’s (2015) model of dialogical and monological speech; in this case, we are dealing with the monological aspect, since it has been argued that the capacities to use monological speech for regulation are crucial for coordinating executive functions in accordance with rules and objectives (Zelazo, 2015).

The above discussion suggests that it is probable that the classification of the children’s PS, in the context of play or the ToL, was not a good methodological tool for measuring their capacity for manipulating different perspectives as the hypotheses of Fernyhough (1996, 2008, 2009) suggests. Nor was it a good measure for testing the broader hypothesis of a general capacity for manipulating semiotic means (Fernyhough & Meins, 2009). This is because, as Luria (2000) said, a word has more than one function (*e.g*., a significance function, a referential function, a regulative function, etc.). In our opinion, what is being measured is the regulative function of language, not the capacity for semiotic manipulation of multiple perspectives as Fernyhough proposed. That is why we found a relationship between the regulative function of language and the planning task with either classification. But we only found a relationship of ToM with the classification of the speech type relative to time (which was not satisfactory). Moreover, it was also clear that the degree of internalization is a problematic measurement since it erases the differences between PS2 and PS3, which are important distinctions, as the results revealed.

The next question would be whether these results imply that the dialogical hypothesis of Fernyhough is wrong and psychological functions are not dialogical at their core. We believe that this is not the case. We believe the problem is the relationship between the ontology (here the dialogical properties of PS) and the methodology (the use of PS coding) since the latter is coherent with the ontology of the regulative properties of PS but not their dialogical ones.

The dialogical nature of the psychological functions is very likely, since there is a lot of empirical and theoretical work demonstrating it in the English-speaking literature (see Berk, 1992; Fernyhough, 1996, 2008, 2009; Winsler, 2009; Wertsch, 1993) and in other regions of the world (see Akhutina, 2003; Akhutina & Pylaeva, 2012; Bibler, 1985; Bodrova et al., 2011; Galperin, 2009, 2021; Luria, 2000; Solovieva et al., 2020). In addition, daily clinical experience shows that the perspectives of others are manifested in their regulation during complex tasks. But we believe this phenomenon is compatible with a non-correlation with ToM. One dimension of the problem, as we mentioned earlier, is that the degree of internalization and private speech are measures of the regulative functions of language, as the time and performance relationship and the type of speech as a function of time suggested. In fact, as we reviewed in the introduction, the executive function theories proposed that ToM and executive functioning are related because ToM tasks need inhibition of the dominant response (*i.e.,* the actual location or actual content in the ToM tasks). Therefore, the relationship between ToM and PS may be of that order.

Another dimension is that domain specific tasks (*i.e*., mental and social understanding) and measurements are required to distinguish the capacities for manipulating multiple perspectives. We recommend that role-playing activities are better suited for evaluating the general capacity to manipulate perspectives. Role-playing activities -- where kids need to predict, explain, or represent the behavior of others -- would be a better fit since they need to adopt the specific perspective of the other with the values and emotional tones appropriate to such perspectives and contexts. They are also a better fit because, according to activity theory, the leading activity at preschool age is role play (Elkonin, 1985; Karpov, 2005). In such play, children manifest actions related to functional roles about which the adults and others have taught them (*i.e*., social norms and adults opinions); and the emotional tones associated with those roles (*e.g*., the charming or repulsive character of someone, at least when it is a more mature role play; Bodrova & Leong, 2006; Bodrova et al., 2011; Elkonin, 1985; González-Moreno, et al., 2014; Karpov, 2005; Solovieva & Quintanar, 2012).

Therefore, one would expect that, as the capacities for role playing develop, the dexterity of ToM would develop, but the degree of internalization would develop too; that the number and richness of roles that children can express in games would correlate with their ToM performance and their degree of internalization; that the degree of expressivity children manifest in role play would correlate with ToM since this would be related to how sensitive they are to others’ perspectives, but also to their degree of internalization since (ex hypothesi) it is necessary for manipulating multiple perspectives; and that the number of voices or perspectives of others manifested in play would relate to ToM performance and their degree of internalization too. Those questions should be addressed in future research.

Because of the above, we favor the idea that PS metrics are better for characterizing the regulative function of language, although we do not discard the idea of a dialogical foundation of the mind. Future studies should explore the use of play for studying the dialogical aspects of PS since inner speech is probably not accessible to introspection to children at this age (Alderson-Day & Fernyhough, 2015).

Finally, another important task for the future would be to take seriously Vygotsky’s ideas of a systemic organization of functions as they change during development (Vygotsky, 2012a, 2012b). In fact, Alderson-Day and Fernyhough’s (2015) dialogical and monological ideas of speech seem to suggest that as speech became internalized, the relationship between PS, executive function, and ToM changes. But further studies should use more comprehensive batteries to test executive functions and ToM tasks, and more coherent forms of evaluating the capacities for manipulating multiple perspectives that language enables.

## Conclusion

In summary, it was acknowledged that as children get older, their ToM and their PS develop, although our sample (*i.e.,* Tlaxcala’s preschool children) suggested that the performance that was expected for ToM did not correspond to what the literature has reported. Even those who were 5.5 to 6.7 years were unable to accomplish all the tasks.

It was also shown that those in first grade of preschool had predominantly more immature forms of regulation compared to their peers in second and third grade. This was clear in the fact that we observed predominantly social speech in first grade, but in second and third grade, self-regulating forms of speech appeared (*e.g.,* PS2 and PS3).

The results also showed that there was not a significant relationship between the degree of internalization and ToM across preschool grades. Nonetheless, when we considered the amount of speech as a function of time (in min), a difference in performance emerged in the maturity of their regulation function. In the third grade of preschool, PS3 was negatively associated with ToM performance. This was in accordance with the dialogical theory of psychological functions of Fernyhough, although it was suggested that PS is a better measure of the regulative function of speech than of the ability to manipulate multiple perspectives. It was advised that role play would be a better task for studying the dexterity of children manipulating multiple perspectives by studying different dimensions of it (*e.g*., the number of roles played, the number of perspectives manifested, the emotional tone expressed in the role, etc.).

## Limitations

One limitation was that the sample was small; therefore analytical power was lower, and the use of parametric methods was impossible. However, robust methods were employed when they were available, or were possible. Another limitation was that we only evaluated executive functions with one task (*i.e*., ToL), so that evaluating the exact relationship between the variables and the other dimensions of executive functioning was not possible. Other studies should include different tasks, such as visual/verbal working memory, inhibition, and cognitive flexibility. Lastly, other studies should evaluate other dimensions of ToM like the precursors of false belief tasks, or the second order tasks of ToM.
